# Pharmacy Students’ Experiences with an Interactive Video Platform to Develop Clinical Reasoning

**DOI:** 10.3390/pharmacy10040083

**Published:** 2022-07-13

**Authors:** Bernadette Cornelison, Adam Baldry, David R. Axon

**Affiliations:** 1 Department of Pharmacy Practice and Science, Tucson Campus, R. Ken Coit College of Pharmacy, The University of Arizona, Tucson, AZ 85721, USA; axon@pharmacy.arizona.edu; 2 Instructional Technologist, Center for Learning Technology, Northwest Campus, Pima Community College, Tucson, AZ 85741, USA; tbaldry@pima.edu

**Keywords:** problem-based learning, teaching, education, pharmacy

## Abstract

Activities used to evaluate clinical reasoning include the use of standardized patients, role play, and case studies. To provide a standardized student experience at a lower cost than a standardized patient, standardized patients were developed using an interactive video platform. The purpose of this article is to report pharmacy students’ perceptions of the interactive video standardized patients used to practice applying clinical reasoning in a self-care therapeutics course. Students participated in the following five methods to assess clinical reasoning: case studies, interactive patient videos, role play, case creation, and Zoom^®^ polls. Four of the five methods (case studies, interactive patient videos, role play, and case creation) were used in small breakout groups consisting of two to three students. Upon completion of the small group work, Zoom^®^ polls assessed the clinical reasoning of the entire class. Students completed a survey that assessed their level of agreement with 17 statements about the course on a five-point Likert scale and 2 questions that asked the students to rank the activities based on their experiences. There were 127 students that took the self-care therapeutics course, and 112 completed the survey (88%). Overall, the students preferred the Zoom^®^ poll activity; however, of the four different methods utilized within the small breakout groups, the findings of our survey indicated that students preferred to receive fully written-out patient cases followed by the interactive patient videos. Additionally, the students thought that the written-out patient cases and interactive patient videos were most efficient for learning and recall. The interactive patient videos may be an alternative activity that allows students to demonstrate and assess their clinical reasoning for each patient case, in addition to seeing how this impacted their patient’s outcome.

## 1. Introduction

Activities incorporating standardized patients, role play, and case studies have been used to teach and/or evaluate clinical reasoning for health sciences students [1]. Human patient simulation training provides students with the opportunity to practice their skills in a high-stress, low-risk environment. This allows the educator to bridge the gap between classroom coursework and “real-life” experiences without compromising patient safety [1,2,3,4]. However, standardized patients can be expensive and difficult to fund [5]. Case studies and role-play learning have been used to engage students to apply the knowledge and skills gained in therapeutic courses to complex clinical cases [6,7]. Through small group discussions, active learning becomes an effective teaching and learning method that requires higher-order thinking skills [8]. Although role play within student groups and case studies is low cost, comprehension is dependent on the facilitator, student, or student groups, which can make it difficult to assess clinical reasoning. In the University of Arizona R. Ken Coit College of Pharmacy (RKCCOP) Doctor of Pharmacy program, the self-care therapeutics course is taught to first-year students in their first semester. Historically, this course has utilized a flipped classroom model by having students engage in patient case activities to reinforce reading content learned from the book before each class. Case studies and role-play activities were provided to the students to practice applying the content to a “real-life” patient. In 2018, students were given an Objective Structured Clinical Examination (OSCE) to assess their knowledge in which the majority of students and facilitators agreed that the OSCE provided a true measure of essential clinical skills required to assess and provide self-care to patients [9].

Videos have been widely used in pharmacy education to prepare students and examiners for OSCEs [10,11,12]. Using virtual patients in pharmacy education is starting to be adopted, but each has its limitations [13,14]. Understanding the value of the OSCE, while appreciating the costs (both direct and indirect) associated with running an OSCE, the course coordinator collaborated with an instructional technology specialist to create standardized patients using an interactive video platform. The purpose of this article is to report pharmacy students’ perceptions of the interactive standardized patient videos used to practice applying clinical reasoning in the RKCCOP self-care therapeutics course.

## 2. Materials and Methods

First-year pharmacy students that were enrolled in the Self Care Therapeutics Course participated in five different methods used to assess clinical reasoning: case studies, interactive patient videos, role play, case creation, and Zoom^®^ polls. During class, students were placed into small breakout groups (2–3 students per group) to work collaboratively through interactive patient videos, fully written-out patient cases, cases that required role play, or patient cases that the students created. All the methods used in the small breakout rooms provided questions that prompted students to provide their clinical reasoning as it relates to the Pharmacists’ Patient Care Process (PCPP) [15]. Students were given approximately 10 min to complete the patient case, simulating the time that a student pharmacist can spend with a patient in the community pharmacy setting. After completing the patient case in the small breakout groups, students returned to the large group in which they were asked the same clinical reasoning questions in the form of a Zoom^®^ poll. This allowed the students to reflect on their answers discussed in small groups as well as provide the professor with a way to identify areas that needed to be reinforced and/or clarified. 

The case studies were fully written-out patient cases followed by questions that required the student to provide their clinical reasoning for choosing their recommendation for the patient. Role play required the students to act out the role of a patient or a pharmacy intern within their small breakout groups. The patient role was provided with a separate document to answer questions from the “pharmacy intern”. The student in the patient role provided prompts to the student in the pharmacy intern role with questions that required the student to provide their clinical reasoning before providing a final recommendation. Case creation required the students to create and complete their own patient cases using a template that resembled the patient cases that were provided by the instructors. 

**Figure 1:**
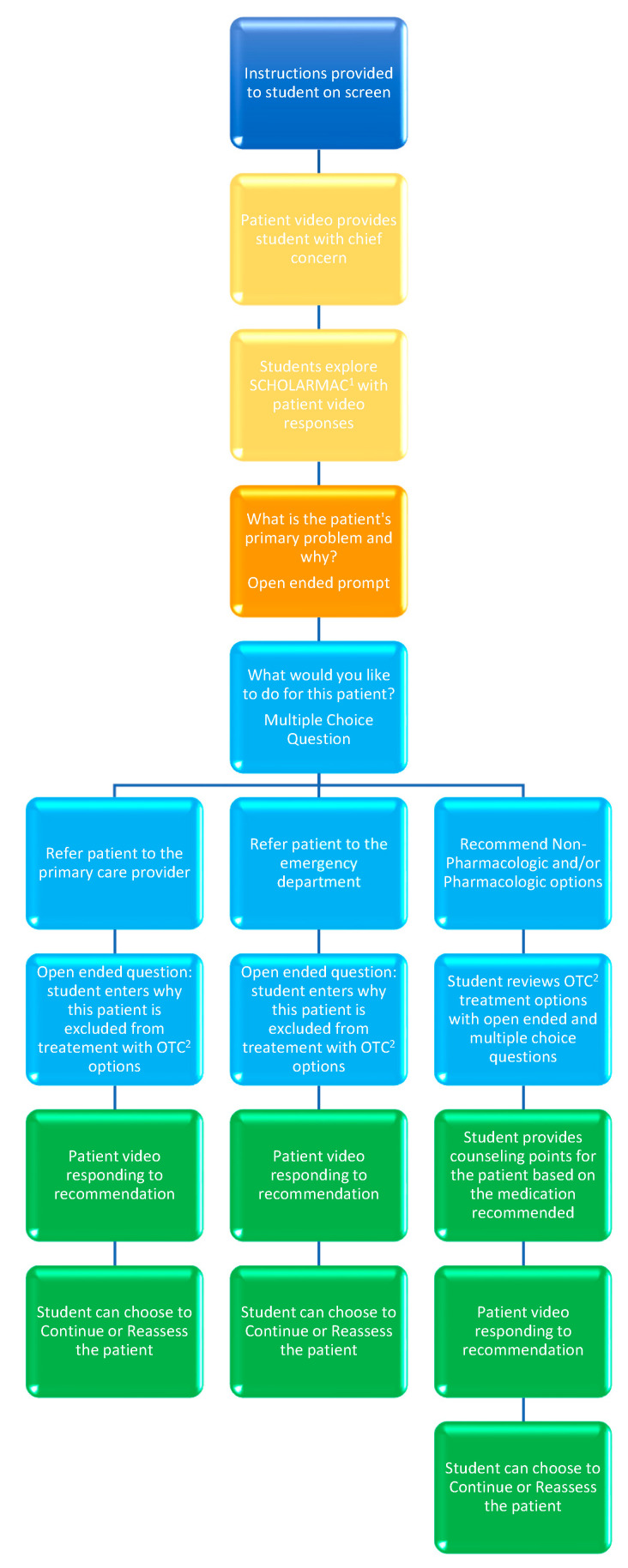
Interactive Patient Videos Flowchart; ^1^ SCHOLARMAC = Symptoms, Characteristics, History, Onset, Location, Aggravating factors, Remitting factors, Medication, Allergies, Conditions; ^2^ OTC = Over-the-Counter.

The interactive patient videos required students to choose their own care path for a patient by using interactive patient videos, multiple-choice questions, and free-response questions (Figure 1). Three decision-making pathways led the students to three different patient outcomes: treatment with an over-the-counter (OTC) medication, referral to primary care provider (PCP), or a referral to the emergency department. 

For each self-care therapeutics topic, short, standardized patient videos were created and uploaded to the interactive video platform. Each of the videos reflected the answers to questions that pharmacy students are trained to ask when evaluating a patient for a self-care recommendation. Additionally, the videos record patient answers in the event that the student chooses the path that may cause the patient harm. For example, a patient experiencing heartburn who has experienced significant weight loss over the previous two months should be recommended to follow up with their primary care provider for a proper workup. If the student chose to treat the patient with an OTC medication, this would delay care for a patient that could potentially have cancer. To reflect a “real-life” scenario, the short patient video would reflect the patient’s dissatisfaction with the inappropriate recommendation. 

Students were required, via a forcing function, to share their clinical reasoning in the form of responses to multiple-choice questions and open-ended questions prior to moving forward in the process of evaluating and assessing the patient. Examples of open-ended questions include: “What is the patient’s primary problem?” and “What about this patient’s clinical presentation led you to your decision?” Multiple-choice questions ask the students what they would like to do next for the patient. For example: “What would you like to do for the patient?” with the following answer options: Refer patient to PCP, refer patient to the emergency department, or recommend OTC options. After the student completes the patient case, the last patient video will reflect the patient’s satisfaction or dissatisfaction with the recommendation provided by the student. After the student sees the patient’s response to their recommendation, the student can choose to continue and complete the patient case (i.e., submit their answers), or they can reassess the patient and work through an alternative pathway.

At the end of the semester, students completed a survey that assessed their level of agreement with 17 statements about the course on a five-point Likert scale and 2 questions that asked the students to rank the activities based on their experiences. The investigators received exempt status for this survey from the University of Arizona Institutional Review Board.

## 3. Results

**Figure 2:**
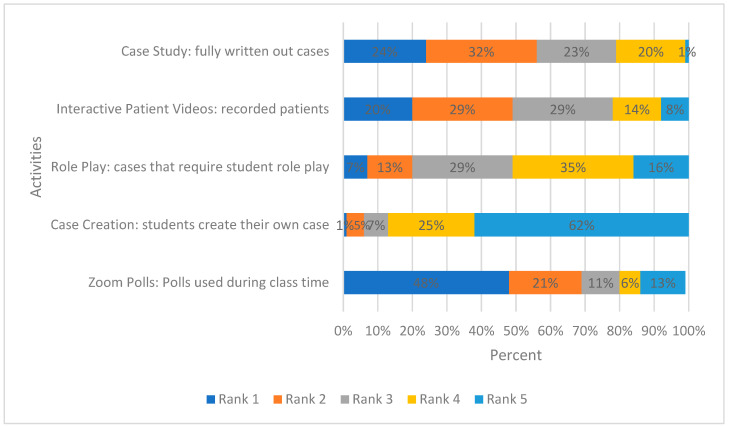
Ranked order of activities students most enjoyed.

There were 127 students that took the self-care therapeutics course, and 112 completed the survey (88%). When asked to rank how much they enjoyed each of the five activities, 48% of students ranked ‘Zoom^®^ polls: polls used during class time’ first. The least enjoyed activity was ‘case creation: students create their own case’ at 1% (Figure 2). 

**Figure 3:**
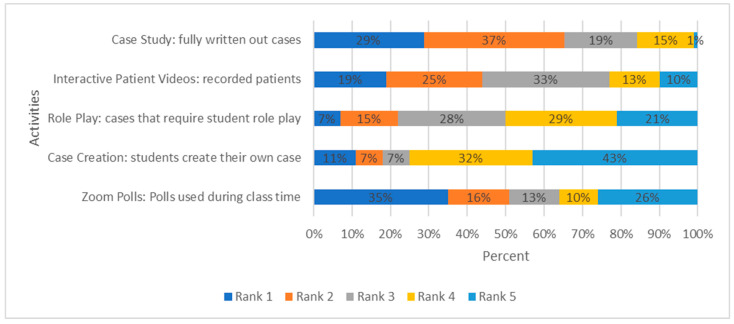
Ranked order of activities students thought were most effective for their learning and ability to recall information.

When asked to rank the activities they thought were most effective for their learning and ability to recall information, 35% ranked ‘Zoom^®^ polls: polls used during class time’ first, 29% ranked ‘case study: fully written out cases’ first, and 19% ranked the interactive patient videos first. Few students ranked ‘case creation: students create their own case’ and ‘role-play activities: cases that require student role play’ first (11% and 7%, respectively, Figure 3). 

When asked to rate their level of agreement with 17 statements about the course on a five-point Likert scale (strongly disagree [1], disagree [2], neither disagree nor agree [3], agree [4], strongly agree [5]), the median scores were strongly agree for one item (I feel I have to prepare for this class in order to do well) and agree for eight items, neither disagree nor agree for seven items, and disagree for one item (My contribution to the breakout group is NOT important). See Table 1 for further information.

Item responses involved a Likert scale with the following options: strongly disagree (1), disagree (2), neither disagree nor agree (3), agree (4), strongly agree (5). 

## 4. Discussion

The Zoom^®^ poll activities provided during the large group discussions were the number one ranked activity of the course. This was anticipated, as this provided the students with feedback on the appropriate clinical reasoning that would lead to the positive patient outcomes after the small group discussions. Of the four different methods utilized to assess clinical reasoning in the small breakout groups (case studies, interactive patient videos, role play, and case creation), students preferred to receive fully written-out patient cases followed by the interactive patient videos. The interactive patient videos differ from the written-out patient cases as the videos require students to critically think about the information that needs to be collected and to determine the information that is pertinent to determining the best approach to providing care for the patient. Thampy et al. discussed the decline in utilizing patient vignettes or case studies to evaluate clinical reasoning, as they only test knowledge acquisition instead of applied knowledge and skill demonstration. Their article further discusses the importance of assessing how learners demonstrate their approach to uncertainty when they apply inappropriate analytic and nonanalytic thinking [16]. The interactive patient videos allow students to demonstrate and assess their clinical reasoning for each patient case, in addition to seeing how this impacted their patient’s outcome. If the patient’s outcome was unsatisfactory, the student can go back and review their clinical reasoning, or they can receive clarification during the large group poll activities. 

Additionally, when provided with a case study or role-play activity in this course, students would tend to immediately implement a plan for the patient without discussing their clinical reasoning with one another. The interactive patient videos incorporate “forcing functions” that require the students to share their clinical reasoning and type them out prior to providing a plan for the patient. Students agreed on survey items that contributed to enhancing one another’s learning in the small group discussions, but neither agreed nor disagreed with how much they enjoyed the small group discussions (Table 1). Students may have found the “forcing functions” that are utilized in the interactive patient videos frustrating as they take time and require additional discussion to take place in the small groups. This requires students to share their thought processes, which can be difficult or uncomfortable for the student if they lack confidence in the subject matter. To alleviate these potential issues that may arise in the small group discussions, students may benefit from completing the interactive patient cases independently and preparing for small group discussions prior to class. 

Excluding the use of other low-cost, technology-based solutions to compare to the interactive patient videos may be a limitation of this study. MyDispense, Pharmacy Simulator, and EHR Go are technology-based platforms that are widely used to provide patient cases to students. MyDispense is an online pharmacy simulation that allows students to develop and practice their dispensing skills [17]. This simulation could be set up to have a patient inquire about a self-care disorder, and the student could recommend or dispense a product to the patient [18]. MyDispense has been used previously at the RKCCOP; however, student reviews were unsatisfactory. Pharmacy Simulator and EHR Go require a cost for student use which is why these platforms were excluded from the study [19,20].

Another limitation of this study is the lack of evaluation of student assessment. To validate this as a valid teaching methodology, comparing student performance in OSCEs or examinations as it relates to the topics would be helpful in determining its usefulness as a tool to assess clinical reasoning.

## 5. Conclusions

The students enjoyed the interactive patient videos; however, they may not have enjoyed the small group breakouts that accompanied the patient videos. Providing the students with the patient videos prior to class may improve student experiences with the interactive patient videos and small group discussions. Further research is warranted to determine the external validity of using interactive patient videos for developing clinical reasoning.

## Figures and Tables

**Table 1:** Student level of agreement with items about the course.

Item	Median (IQR) ^1^
I spend time studying before class in order to be more prepared	4 (1)
I feel I have to prepare for this class in order to do well	5 (1)
I contribute to my breakout group members’ learning	4 (1)
My contribution to the breakout group is NOT important	2 (1.25)
My breakout group expects me to assist them in their learning	4 (1)
I am accountable for my breakout group’s learning	4 (1)
I am proud of my ability to assist my classmates in their learning during breakout	4 (1)
I need to contribute to the breakout group’s learning	4 (1)
I enjoy the breakout room activities	3 (2)
I learn better in a team setting (such as breakout room setting)	3 (2)
I think that breakout room learning activities are an effective approach to learning	3 (1)
I do not like to work in groups	3 (2)
The breakout group learning activities were fun	3 (1)
The breakout group learning activities are a waste of time	3 (2)
I think the breakout group learning helped me improve my grade	3 (1.25)
I have a positive attitude towards breakout group learning activities	4 (1)
I have had a good experience with the breakout group learning activities	4 (1)

^1^ IQR = interquartile range.

## Data Availability

Data are available from the corresponding author upon reasonable request.
